# The Use of Closed-Circuit Television and Video in Suicide Prevention: Narrative Review and Future Directions

**DOI:** 10.2196/27663

**Published:** 2021-05-07

**Authors:** Sandersan Onie, Xun Li, Morgan Liang, Arcot Sowmya, Mark Erik Larsen

**Affiliations:** 1 Black Dog Institute University of New South Wales, Sydney Sydney Australia; 2 School of Computer Science and Engineering University of New South Wales, Sydney Sydney Australia

**Keywords:** suicide, suicide prevention, CCTV, video, computer vision, machine learning

## Abstract

**Background:**

Suicide is a recognized public health issue, with approximately 800,000 people dying by suicide each year. Among the different technologies used in suicide research, closed-circuit television (CCTV) and video have been used for a wide array of applications, including assessing crisis behaviors at metro stations, and using computer vision to identify a suicide attempt in progress. However, there has been no review of suicide research and interventions using CCTV and video.

**Objective:**

The objective of this study was to review the literature to understand how CCTV and video data have been used in understanding and preventing suicide. Furthermore, to more fully capture progress in the field, we report on an ongoing study to respond to an identified gap in the narrative review, by using a computer vision–based system to identify behaviors prior to a suicide attempt.

**Methods:**

We conducted a search using the keywords “suicide,” “cctv,” and “video” on PubMed, Inspec, and Web of Science. We included any studies which used CCTV or video footage to understand or prevent suicide. If a study fell into our area of interest, we included it regardless of the quality as our goal was to understand the scope of how CCTV and video had been used rather than quantify any specific effect size, but we noted the shortcomings in their design and analyses when discussing the studies.

**Results:**

The review found that CCTV and video have primarily been used in 3 ways: (1) to identify risk factors for suicide (eg, inferring depression from facial expressions), (2) understanding suicide after an attempt (eg, forensic applications), and (3) as part of an intervention (eg, using computer vision and automated systems to identify if a suicide attempt is in progress). Furthermore, work in progress demonstrates how we can identify behaviors prior to an attempt at a hotspot, an important gap identified by papers in the literature.

**Conclusions:**

Thus far, CCTV and video have been used in a wide array of applications, most notably in designing automated detection systems, with the field heading toward an automated detection system for early intervention. Despite many challenges, we show promising progress in developing an automated detection system for preattempt behaviors, which may allow for early intervention.

## Introduction

Suicide is a recognized public health priority, with approximately 800,000 people dying by suicide each year [1]. The use of different means of suicide can vary by geographic region, with public means such as jumping from a height being more common in certain areas [2]. Data from reviews of coronial records in the UK suggest approximately 30% of all suicide deaths occur in public places [3]. Suicides in public places can attract media attention, potentially introducing a degree of “notoriety” about a specific location, and thus increase its frequency of use [4]. Furthermore, these incidents can adversely affect bystanders [5]. Thus, efforts to prevent suicide in public places are of particular importance.

A range of suicide prevention initiatives in public places have been evaluated, with the use of CCTV cameras being proposed as a means to increase the likelihood of a third-party intervention [6], for example, by initiating a police call-out after climbing a safety fence [7]. Studies from the metro/underground railway settings have retrospectively analyzed behaviors which precede a suicide attempt, with observable behaviors being identified [8,9]. These studies have further suggested that it may be possible to automatically detect behaviors prior to a suicide attempt, potentially allowing for earlier intervention and the interruption of a suicide attempt.

This paper builds on these foundations by addressing 2 core aims. First, it provides a scoping review on the existing use of CCTV and other video data to understand and prevent suicide. Second, it reports progress on an ongoing study to respond to an identified gap in this literature, by using a computer vision–based system to identify behaviors prior to a suicide attempt.

## Methods

Searches of the literature were performed using the PubMed, Inspec, and Web of Science databases, as pilot searches indicated that these databases provided unique literature from medical and computer science publications. Searches were performed using the terms “(video OR cctv) AND suicid*” on all fields. Searches were performed on August 26, 2020, and included all entries from conception to that date.

We sought to understand the broad use of video, and therefore included studies which (1) related to understanding suicide or suicide prevention; (2) described the use, analysis, or consideration of video footage or CCTV; and (3) related to the understanding of risk factors for suicide, used video monitoring to detect suicide or suicide risk, or used video data pertaining to individuals. We excluded studies of video gaming (with no use of video data), video-based interventions (ie, showing a video to a person or group, or portrayal of suicide in video), or video-based telehealth interventions, suicide bombing, music videos, video vignettes, nonhuman subjects, or EEG-CCTV. Furthermore, we excluded any commentaries and opinion pieces without data or studies described, or studies which were not reported in English.

Abstracts were independently screened for eligibility by 2 reviewers (SO and ML), with disagreements resolved by discussion until consensus was achieved. Eligible papers were then downloaded for full-text review, and 1 reviewer (SO) extracted a narrative summary of the reported use of video data.

## Results

### Database Search and Review of Articles Retrieved

**Figure 1:**
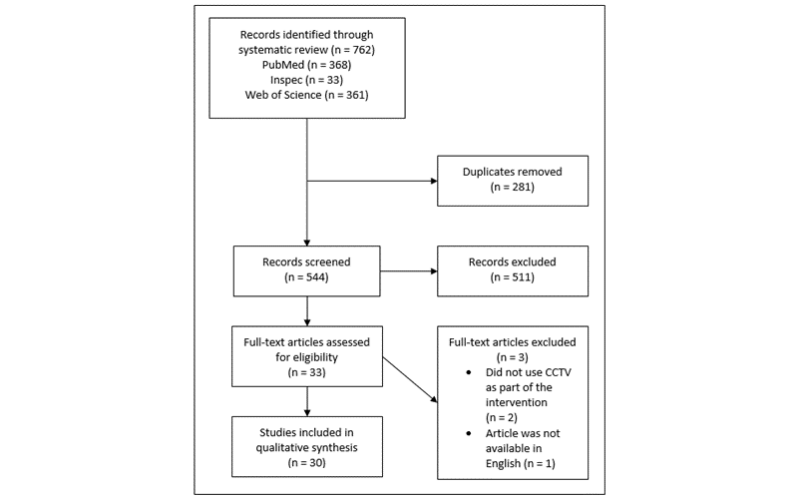
Flowchart of Study Selection.

Database searches identified 544 unique articles to screen, as indicated in Figure 1. Following review of the title and abstracts, 33 articles were downloaded for full-text review. Two studies were excluded as they did not report relevant use of video or CCTV data, and 1 was excluded as it was not available in English. The remaining 30 articles were retained for the narrative synthesis.

In the review, we identified 3 main categories of papers corresponding to their temporal relationship with suicidal behavior. These are characterized as (1) studies that use CCTV and video to understand risk factors for suicide (6 papers); (2) studies that describe and evaluate interventions using CCTV or video footage (16 papers); and (3) studies that use CCTV or video footage to understand suicide after an attempt (8 papers). In this section, we discuss the findings of the review in these categories; however, we discuss the studies within the second category, that is, using CCTV or video as an intervention, last due to its length.

As many of the studies described below use machine learning and computer vision techniques to obtain desired results, for example, predicting depression from facial cues, a short introduction to relevant computer vision techniques is provided below.

### Computer Vision Overview

In this section we describe some basic concepts in computer vision to aid understanding of the described studies. Computer vision is a subfield of artificial intelligence that trains computers to interpret and develop a high-level understanding of the visual world. It seeks to replicate the workings of human perception by extracting semantic information from image data. It aims to automate the time-consuming human processes, analyzing a much larger volume of data with real-time efficiency. Two examples of its application discussed in this paper include pedestrian tracking, in which the algorithm is able to identify people within a scene and interpret their behavior, as well as facial analysis, in which the algorithm detects key points on a face and attempts to infer emotional states from the configuration of these points.

Applications of computer vision techniques in analyzing video footage to understand human behaviors in a natural environment (such as outdoor scenes captured by CCTV cameras) have typically followed a processing pipeline involving object detection, object tracking, and action recognition. Object detection refers to the ability to recognize and locate objects of interest in each frame of a video [10]. Object tracking is the process of locating the object of interest in multiple sequential frames over time [11]. Action recognition aims to identify the actions and goals of the objects of interest from a series of observations [12]. There are numerous algorithms and methods available to achieve a single goal, introducing the need for experimentation, which involves applying a variety of different methods to the same data and measuring the outputs against relevant performance metrics.

Paramount to the success of the aforementioned pipeline is the ability to gather a considerable amount of relevant data for a given task. This relates to the fact that these algorithms can be “taught” the required task by training them with annotated data, which is denoted as supervised learning in the field of machine learning. The data are annotated with labels that denote the location of the object of interest within the image and other semantic information about the object in the video frame. These annotations also help in adapting the models to the characteristics of the CCTV data such as camera environment, camera angle, and key object features. Overall, this increases the accuracy of the algorithm on the task at hand, for example, identification, tracking as well as action recognition.

In the following sections, we note specific names of algorithms for thoroughness. However, we do not go into their technical details, and the names are mentioned alongside their functionality.

### Understanding Suicide Risk Factors

As described above, the first category of studies identified in this review related to the use of video and CCTV data to understand suicide risk factors. Within this category, 2 main types of studies were identified. The first analyzed videos of individuals who died by suicide, in order to understand risk factors. In one study, researchers searched video footage of suicides posted on Facebook in Bangladesh to understand the circumstances that may have contributed to the suicides [13]. The authors found 19 videos, predominantly of male students. Hanging was the most frequently used means, with relationship problems, academic stress, and mental health disorders being commonly identified causes.

The second type of study attempted to infer depression from facial cues using both human and computer vision. For example, in one study [14], the authors attempted to assess whether humans are able to perceive depression in another person at both a conscious and a physiological level. Participants viewed videos of individuals with depression while various physiological responses of the viewer were recorded (eg, galvanic skin response, temperature, and heart rate). The study found that despite not being able to consciously determine whether the person they were viewing had depression, the viewer’s physiological response differed as a function of that person’s depressive state.

Beyond manually assessing footage, automatic video analysis from the field of computer vision and machine learning has been used. Many studies in this category attempt to identify depression from facial cues [15-18]. For example, in one study [17] participants were shown a sad video, a neutral video, and a text to read and were interviewed while their facial expressions were analyzed using a Microsoft Kinect camera. The study found that when watching a neutral video, and using information primarily from the eyebrows and mouth, it was possible to predict the presence of depression above chance level (86.8% for females and 79.4% for males).

While different studies adopted different methodological approaches, the critical difference between the reported studies is in the experimentation protocols adopted when applying various computer vision algorithms and approaches to best achieve the desired outcome. For example, to infer depression from facial expressions, Girard et al [19] used video to locate 66 facial points and extract facial features surrounding those points, converting the visual stimuli into features. After extracting this information, the authors used a dimension-reduction approach to reduce the amount of data processed. Following that, a support vector machine with a radial basis function kernel classifier (a machine learning algorithm that classifies data into categories) was trained using the data, which was then able to detect depression comparable to manual coding of facial features present in individuals with depression. In another approach, Scherer et al [20] used MultiSense, a system built upon a combination of a variety of head and facial tracking algorithms, to analyze the following behaviors: head gaze (orientation), eye gaze, smile intensity, and smile duration. Features such as gaze direction, duration, and intensity of smiles were measured and their correlation with distress, depression, anxiety, and posttraumatic stress disorder analyzed. Therefore, different algorithms and approaches can be used for the same goal, with differing rates of success.

### Understanding Suicide After an Attempt

In the third category of using videos and CCTV to understand suicide after an attempt, all the studies related to forensic examinations. As a result of this, many of these studies are quite graphic in nature, and while we attempt to describe these studies as simply as possible, there may be text that readers may find disturbing. If this is the case, please skip over this to the next section.

Most videos sought to understand the sequence of bodily reactions from the suicide attempt to eventual death, called the *agonal sequence*. Understanding these sequences can help forensic scientists to retrospectively determine details surrounding an individual’s death. By far, the most commonly reported means of suicide was hanging, in which researchers viewed recorded hangings and analyzed the process by which the individual dies [21-24]. All the studies noted that despite hanging being the most common method of suicide, there is little known about the sequence of events leading to eventual death. In all studies, the researchers viewed the hangings and noted how long it took for certain events to occur (eg, loss of consciousness, convulsions, rigidities).

In addition to death by hanging, some studies examined other cases of suicide to understand details of interest. For example, in one study, forensic experts studied a case of self-immolation [25]. In another, researchers studied the death of an individual at a Swiss right-to-die organization by oxygen deprivation [26]. The organization typically used barbiturates, and the researchers were assessing the feasibility of using oxygen deprivation, noting ways to improve the process.

Two forensic studies highlighted the importance of CCTV and video footage in determining whether a death was due to homicide or suicide. In one study, an individual presented with stab wounds to the neck, which was initially assessed as a homicide. However, the authors noted that due to the presence of CCTV, they were able to conclude that the individual had died by suicide [25]. In another study, an individual was found to have died due to impact by a motor vehicle [26]. While the individual presented with self-harm scars and prior substance use, the CCTV of a neighboring business was able to definitively conclude that this was in fact a suicide. This is incredibly important as certain deaths are difficult to rule as suicide, and thus CCTV can enable us to determine them as such.

### Describing and Evaluating Interventions Using CCTV or Video Footage

In the second category of studies, researchers sought to develop new ways to utilize CCTV and monitoring to identify persons in crisis or to allow an intervention during an attempt. This has been identified as 1 of 3 key interventions outlined in a meta-analysis of suicide prevention interventions at hotspots (ie, increasing the possibility of an intervention by a third party) [6]. This often takes the form of CCTV monitoring with or without an accompanying automated alert system or analyzing past CCTV footage to understand crisis behaviors.

Studies have shown that having human-monitored CCTV cameras at a site may reduce the incidence of suicide. For example, in prisons where hangings occur, a study compared the incidence of various behaviors in areas covered and not covered by CCTV and found that there were fewer incidents of suicides in monitored areas [27]. However, this conclusion is undermined by the fact that hangings may most likely occur in areas that do not have CCTV cameras (eg, cells). In another study, the authors investigated the effect of CCTV at metro stations in the state of Victoria in Australia [28]. The authors found that CCTV was able to reduce the incidence of suicides. It is not known whether this is due to individuals being more wary in the presence of cameras or the station staff being able to intervene earlier. Finally, another study investigated the efficacy of a virtual monitoring system for patients with psychiatric disorders in a general hospital setting, in which 1 person was able to monitor up to 10 rooms with patients. While the study results were not conclusive, it appeared that the system allowed staff to monitor the safety of more patients at the same time [29].

One key factor associated with the systems described above is that they rely heavily on manual monitoring. Therefore, automated or semiautomated systems have been developed and tested. For example, a study examined a bridge in Seoul which used manual CCTV monitoring along with infrared motion sensors [30]. The sensors would detect when a person climbs over the fencing or jumps into the water below, alerting the guards stationed close by.

Another similar approach to automated monitoring is to draw regions of interest in a static CCTV scene. For example, Mukherjee and Ghosh [31] described an automated detection system in a metro station. The system detects movement within a predefined region of the video footage. Any movement in that region beyond the arrival of trains would send an alert to station staff. Similarly, 2 other studies described a similar approach at a cliff located in Sydney, Australia [5,7]. Unlike the metro station, this outdoor site was much larger and with extremely uneven geography, leading to less complete CCTV coverage. The region beyond the fence is specified as a region of interest and if movement is detected on the wrong side of the fence, an alarm is triggered at a monitoring station. While this approach of identifying static regions of interest within scenes has allowed the successful delivery of suicide prevention interventions, this method is only effective in locations where the means of suicide relied on individuals crossing over a boundary or a fence (eg, a cliff or metro station).

Another form of automated detection uses behavioral identification using automated vision methods to detect a hanging. Rather than using motion sensors or an outlined region of interest in the CCTV scene, these methods use computer vision analysis to detect a person in the scene, their limbs, as well as when the position and movement of those limbs indicate a suicide attempt by hanging [32-36]. In one study, the authors developed a video surveillance system to detect a suicide attempt by hanging using color cameras that can also detect depth (red, green, blue—depth cameras; RGB-D) to understand the three-dimensional positions of body joints [33]. Hand-crafted features are extracted based on the joint positions. More specifically, pair-wise joint distances within each frame and between 2 consecutive frames are used as feature vectors for classification. A linear discriminant classifier (LDC) was trained to classify 2 groups of actions: nonsuspicious actions (such as move, sit, wear clothes) and suicide by hanging. However, the training data set was only based on simulated video recordings taken in a room environment rather than genuine, unplanned events. Similar methods have also been adopted in other studies [20,36].

With the increased emergence of multipurpose intelligent computer vision systems, a paper proposed hierarchical evaluation metrics to evaluate such a system at metro stations, using three dimensions of performance [37]. The first dimension is object detection, which is the system’s ability to accurately detect an object of interest (eg, a person or a bicycle). This is important to prevent triggering false alarms if a nonhuman object is identified within a defined region of interest. The second dimension is object tracking, which is whether the object of interest is able to be tracked within the scene across time and different image frames. Within this object tracking, there are 3 factors: trajectory similarity, which identifies the path taken within the frame; ID changing, which is whether an object is consistently identified as the same object during tracking; and latency, which is how quickly the system can track the object. Finally, the third performance dimension is context awareness, which is how accurately the system is able to identify the situation (eg, a person currently in an unsafe location, such as walking on railway tracks).

However, even when using automated systems, the ability to intervene is limited to when an attempt is made or a very short period prior to an event, such as entering an unsafe location. As a previous study [33] notes, the next step forward is to find ways to detect presuicidal behaviors to reach out to people in crisis prior to an attempt, and a necessary step is to first and foremost identify these behaviors. By investigating predictive behaviors, a third party may be able to intervene earlier or more quickly, which could greatly reduce the incidence of suicide. Thus far, there have been only 3 studies investigating such predictive behaviors, including 1 study using videos from social media, and 2 studies at metro stations.

In the first study, the authors used videos of livestreamed suicides posted on social media and observed behaviors preceding the suicide [38]. In particular, the authors investigated 3 behavioral markers: verbal markers to investigate whether the individual talks more about self-harm and uses more negative language; acoustic markers to investigate whether pitch and pauses in speech are indicative of suicide; and visual behavior markers to investigate whether there are any visually identifiable markers (eg, frequently shifting eye gaze or pose changes). The analysis revealed that frequent silences, slouched shoulders, and use of profanity are indicative of elevated suicide risk.

In one study, over the course of 2 experiments, the authors attempted to identify behaviors indicative of a subsequent suicide attempt at metro stations, and tested how well these behaviors predict an attempt [8]. In the first experiment, the authors identified key behaviors by having 2-3 observers code both easily observable behaviors (eg, standing near the edge of the platform) and those behaviors requiring interpretation (eg, looking depressed). In the second experiment, the authors identified several behaviors by analyzing 5-minute segments of a video footage, with or without an attempt at the end, to check whether these behaviors were indeed present in individuals who were in crisis. The results suggested that 2 behaviors (pacing back and forth from the edge of the platform, and leaving belongings on the platform) could identify 24% of the attempters with no false positives. This suggests that there are indeed crisis behaviors that can be observed, and within 5 minutes prior to an attempt.

Another study [9] expanded on the findings from the aforementioned study [8] and used semistructured interviews and questionnaires administered to railway staff in addition to CCTV footage to identify these potential indicator behaviors. The process identified 5 main behaviors: station hopping and platform switching, limited contact with people, allowing trains to pass by, where they stood on the platform, and repetitive behaviors such as walking up and down the length of the platform.

CCTV and videos have been used in a variety of ways to understand and to prevent suicide. Studies provide evidence that manual CCTV monitoring may in fact reduce incidence of suicide in various settings, such as in a hospital or in metro stations. Newer approaches utilize automated systems, such as using thermal motion sensors or defining a region of interest in CCTV scenes, while others utilize computer vision to detect behaviors indicative of a suicide attempt by hanging [20,33,36]. Finally, others have examined crisis behaviors as a starting point to develop interventions that are able to reach out even earlier [8,9].

## Discussion

### Interim Findings

This review shows that CCTV and video data are an important addition in understanding and preventing suicide. This review focused on the peer-reviewed literature, and did not include gray literature such as reports, working papers, government documents, white papers, and evaluation. Future reviews may seek to additionally include such gray literature. Nevertheless, we found that computer vision was able to objectively identify depression, an important risk factor for suicide, above chance level. This may potentially provide new avenues for screening or triage, given its objectivity and ability to process large amounts of data at once.

Other uses help forensic scientists understand how death occurs during certain means of suicide, and critically was able to provide evidence that helped rule distinct incidents as suicides. One of the challenges in understanding suicide is ruling when it has occurred. By incorporating CCTV and video into this process, we are able to better understand when and how suicides occur. Without fully understanding the means by which people die by suicide, we are unable to prevent it. CCTV and video are able to help with this process.

Finally, CCTV has been used in preventing suicide, with novel approaches being developed or investigated. These approaches include the use of thermal sensors, defining a region of interest in the scene, and more recently using computer vision. Thus far, while studies have been able to identify behaviors of a hanging attempt using computer vision, and some other studies have been able to identify signs of a subsequent attempt [8,9], there have been no studies published that identify crisis behaviors and attempt to identify them using computer vision.

### Current Work: Integration of CCTV and Machine Learning at Hotspots

#### Aims

As identified in the preceding review of the literature, the use of CCTV has the potential to play a crucial role in suicide prevention at certain locations. While computer vision has been used to identify hanging, it has not yet been used to identify presuicidal behaviors in public places such as suicide hotspots. Critically, while available systems are able to send an alert once an attempt is in progress (eg, after climbing a fence or entering the railway tracks), our work aims to detect early behavioral indicators that may precede a suicide attempt, which will may therefore allow more time for intervention and prevention. In this section, we briefly discuss our work in progress, where we use CCTV data and machine learning to identify crisis behaviors at hotspots. We present our approach to fill this gap for automated presuicide action detection.

#### Setting

Data are collected from a coastal tourist destination in Sydney, Australia. In 2010, the local council, along with several partners, implemented a multilevel self-harm and suicide prevention strategy, which included installing various types of fencing, improving amenities, installing signage and hotline booths, as well as installing CCTV cameras. At this location, there are 3 key types of cameras: static RGB color cameras; pan, tilt, and zoon (PTZ) RGB color cameras; and thermal cameras. The thermal cameras primarily cover the fences where people often cross over and thus have a much wider range. Further, given that many incidents happen at night, thermal cameras are used to trigger an alert if an individual crosses the border, by defining a region of interest. Once an alert has been sent, the police are notified. During daytime, PTZ RGB cameras can be used to locate the person of interest. This study has been approved by the UNSW Human Research Ethics Committee.

#### Methodology

To identify signs of a person in crisis, we propose conducting a human coding study similar to previous studies [8,9] and adapting the findings to a computer vision–based framework. The identification of these behaviors is critical to the design and structure of the information pipeline. In earlier works from metro settings [8,9], several key behaviors were identified, including pacing back and forth from the edge, neatly placing shoes and bags on the platform, and standing at the end of the platform where the train would approach. One challenge this poses is that while as humans we can identify these patterns as noted by Mishara and colleagues [8], in computer vision identifying different types of behaviors may require different approaches. Within each behavior type, the underlying system has different levels of complexity. For instance, placing items on the platform would require the algorithm to identify that the person has interacted with another, separate object. Methods to detect pacing back and forth from the edge, by contrast, would require pedestrian tracking along with formulating a trajectory—a path illustrating the object’s movement within the scene. To recognize multiple humans’ actions in untrimmed videos from CCTV cameras, 3 levels of vision processing are required: human detection (low-level vision), human tracking (intermediate-level vision), and behavior understanding methods (high-level vision) [39]. To identify certain high risk and potential indicator behaviors, a human coding study is required to provide annotated data for training the subsequent machine learning processes.

#### Behavioral Annotation

Incidents where an individual has climbed the safety fence and triggered an alarm will be retrospectively identified from site incident logs. Incidents which are noted as a noncrisis incident (eg, on the wrong side of the fence to take photos) will be excluded. Recordings of incidents will be extracted, and individuals traced back, as far as possible, to their entry to the site. Based on the 2 human coding studies [8,9] that previously reported analyses of 60 and 16 clips, respectively, we aim to analyze 16 incident clips. These will be matched by an equal number and duration of control clips of routine behaviors.

Following again the 2 studies identifying crisis behaviors at metro stations [8,9], relevant behaviors which have been identified from the metro setting will be generalized to form a codebook of behaviors which are likely to be relevant to this setting. Two researchers will review each clip and note any observable behaviors, their time of onset, and duration. Whether a behavior is thought to be positively or negatively associated with distress will also be recorded. These behavioral annotations will then be used as training data for the computer vision algorithms.

#### Behavior Identification Through Computer Vision

**Figure 2:**
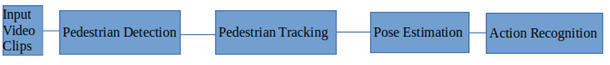
Proposed Pipeline for Footage Processing.

In this stage, we aim to develop an automated system that can detect specific human behaviors in real time for suicide prevention. The video clips will be fed through a data pipeline consisting of 4 layers of function modules, each with their own goal: the first is a pedestrian detection module to detect objects and persons in the clip; the second is a pedestrian tracking module to track the individual in the scene; the third is a pose estimation module to outline the location and configuration of the human joints and limbs (similar to that used by [32-36] to identify hanging); and the fourth is action recognition which interprets the configuration and motion of the joints and limbs to infer behavior (Figure 2). Using this approach, we are able to cover a wide range of behaviors. For example, if the individual is taking a photograph (a hypothetical routine behavior), the action recognition module should be able to identify it and eliminate that person from interest, or if the person walks back and forth from the fence, a hypothetical crisis behavior, then tracking should be able to capture this back-and-forth trajectory. We briefly discuss the 4 stages, noting the selected algorithms. These algorithms were not selected on the basis of their performance alone, as each camera, location, and context is different. Rather it is the result of extensive experimentation, the full details of which are out of the scope of this review.

**Figure 3:**
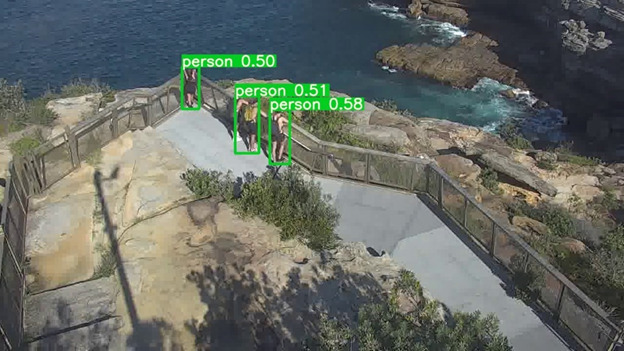
Pedestrian Detection Output Prior To Finetuning. Only three of the four individuals were detected, and all had low confidence values.

**Figure 4:**
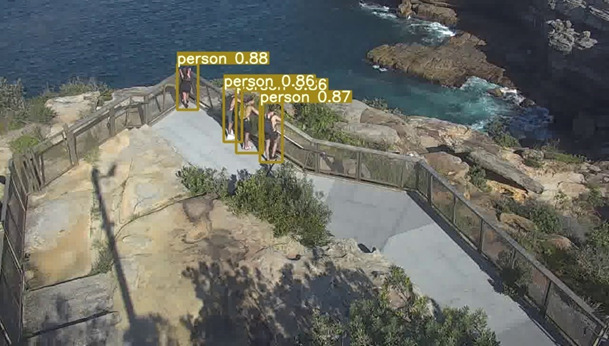
Pedestrian Detection Output using the Fine-tuned Algorithm. All four pedestrians were identified, and with high confidence values.

In the first level, the algorithm should detect pedestrians or objects of interest. In order to do this, we use a deep learning–based object detector named YOLOv5 (You Only Look Once, version 5; [40]), as it provides a good balance between accuracy and processing speed in our context and reflects the most recent progress in the field. As noted above, these algorithms require training, and the algorithm is pretrained on a large public data set named MSCOCO [41]. However, the camera parameters such as angle and depth in the public data set differ largely from those of the cameras used at the site. This results in large differences in the visual appearance of pedestrians in the scene (such as shape and scale); therefore, we further trained (fine-tuned) the model using annotated footage from the location for customized pedestrian detection. The algorithm output before retraining on our data set is illustrated in Figure 3, and Figure 4 illustrates the output after retraining on our data set. In both figures, pedestrians are indicated using bounding boxes, which are the smallest possible enclosing box around the individual. A confidence score (0-1) is shown above each pedestrian, indicating the level of confidence that the algorithm has detected a person within the box. As can be observed, the algorithm without fine-tuning (Figure 3) identified 3 individuals with a relatively low confidence (0.50-0.58), whereas after fine-tuning by training the algorithm with an annotated footage from this location (Figure 4) 4 individuals were correctly identified with higher confidence (0.86-0.88).

The output of the pedestrian detection module is then used as the input of the pedestrian tracking module that can track the same individual through multiple frames of footage. To that end, we chose a classical detection-based tracking method, named DeepSORT [42], which is a tracking algorithm with good speed and accuracy. The inputs to this module are the detected pedestrian regions (in the form of bounding boxes). The tracking algorithm assigns a unique identifier to each pedestrian, and tracks each target throughout the duration when it is visible in the scene. A challenge in tracking is ID switching, which is when 2 people overlap and thus 1 person is occluded in the camera’s view, and the algorithm switches their initially allocated IDs. The current DeepSORT algorithm is able to alleviate such problems by including association metrics that use similarities between appearance features extracted by deep neural networks.

**Figure 5:**
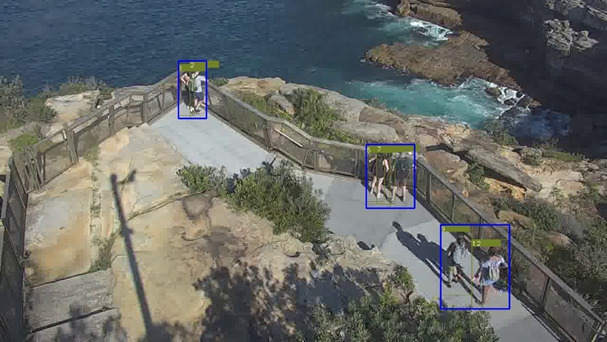
Pedestrian Tracking with Group Functionality. Three groups of people are detected in the scene: (1,2), (5,7), and (9,12). Each pedestrian is represented by a unique ID, and groups enclosed by blue rectangles.

At this stage, we also include 2 other modules, namely, grouping and trajectory analysis. We include grouping analysis, as individuals who travel in a group are hypothesized to have a substantially lower risk of being in crisis compared with individuals. These groupings are calculated by measuring how far apart individuals are from each other and whether they travel at similar speeds, as well as their scale differences. If the algorithm perceives that they are in the group, a bounding box is used to group them together—see Figure 5 for an example output.

**Figure 6:**
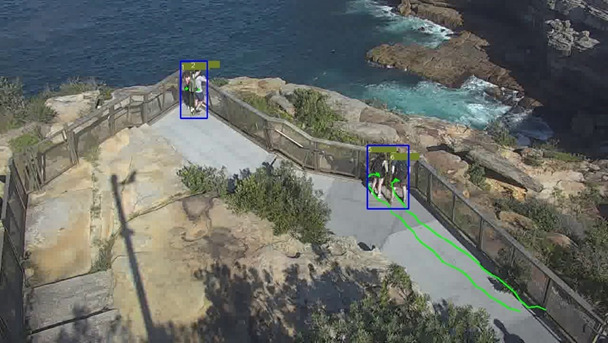
Pedestrian Tracking with Trajectory Functionality. Pedestrians 1 and 2 have been stationary, so the trajectories are collapsed to green dots. Pedestrians 5 and 6 have walked along the fence line, as shown by the green path.

A second functionality for this module is trajectory analysis. The trajectory for each pedestrian is stored for a predefined duration. This functionality is included as past studies investigating crisis behaviors have found that walking or pacing back and forth may be indicative of a future attempt. Figure 6 shows example trajectories for groups of stationary and walking individuals.

**Figure 7:**
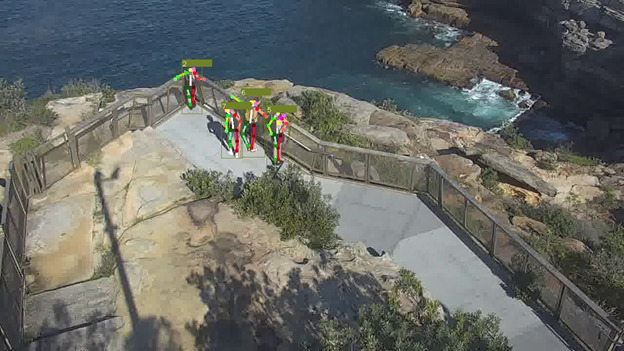
Pose Estimation Module Output. The joint locations and limbs are identified by a pose estimation model. For better visibility, the other visualizations for grouping and trajectory analysis have been omitted.

The third component of our pipeline is pose estimation, which is the estimation of the joint positions in the human body and connecting them. Using information from the previous modules, the pose estimation module analyses the areas within the bounding boxes and estimates the position of various joints. This is shown as stick-figure outlines overlaid on top of the individual (Figure 7). The accuracy of the pose estimation algorithm depends on the image quality, with a closer view, of higher resolution, resulting in more accurate estimates.

In previous modules, each pedestrian has been detected, tracked, and their joints located. In the final module, pose-based features are extracted from each individual pedestrian over a time window and used to detect behavior. Once features have been extracted from video footage containing different actions, they are used to train machine learning–based classifiers to classify the actions, such as support vector machines [43], random forests [44], and multilayer perceptrons [45].

We have thus far integrated 4 preliminary actions or behaviors: standing, walking, taking a photo, and waving. These are actions which are readily observed in routine footage and will be augmented with actions identified through the behavioral annotation of incident footage upon completion of data collection.

**Figure 8:**
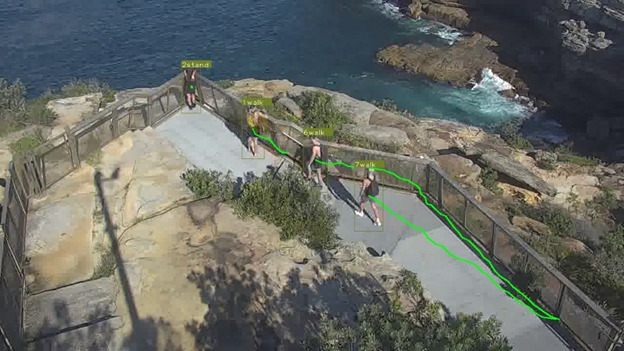
Example Output of the Analysis Pipeline. Individual pedestrians are shown with their unique IDs, recent trajectories, and action class (stand/walk). No groups were identified in this frame. Pose estimation has been omitted to improve visibility of the other outputs.

**Figure 9:**
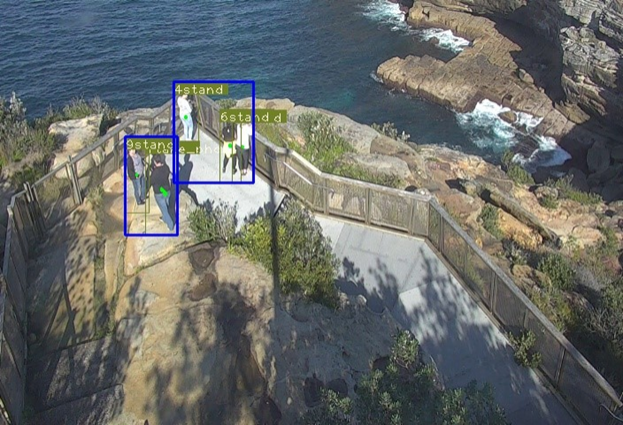
Example Output of the Analysis Pipeline 2. In this frame, five pedestrians within two groups have been identified. Their actions have been identified, with four individuals standing (“stand”) and one individual taking a photo (“take photo”). Their trajectories (in the form of dots and short lines at the centre of each person) indicate that they have remained in their current positions for the recent duration.

In summary, our current pipeline is able to detect an individual, track, and illustrate his/her path through the scene, identify groups, estimate the configuration of his/her limbs, and infer behaviors. Figures 8 and 9 show visual summaries of the overall analysis pipeline.

#### Thermal Cameras

**Figure 10:**
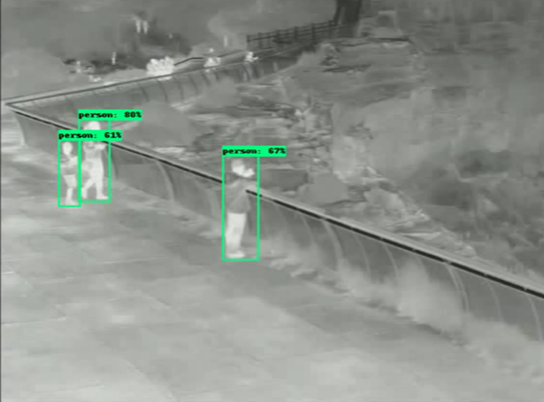
Pedestrian Identification using Thermal Cameras. The output of the EfficientDet pedestrian detection algorithm using data from a thermal camera. Individuals in the foreground are shown within bounding boxes with confidence levels between 61-80%.

The pipeline described in the previous section was implemented using footage from the RGB color cameras. However, a number of cameras at the site are thermal based, and the algorithms cannot be directly applied to these cameras. Therefore, a similar pipeline is also currently being developed for the thermal cameras. To date, pedestrian detection has been implemented using the EfficientDet algorithm [46]. This algorithm was selected based on its comparatively high accuracy performance compared with other available object detection algorithms on thermal data. Similar to YOLOv5 [40], this algorithm is pretrained on the large MSCOCO [41] public data set, and Figure 10 illustrates the output. Further data collection and annotation are required to allow fine-tuning to increase the accuracy of the algorithm (similar to the improvement shown between Figures 3 and 4).

**Figure 11:**
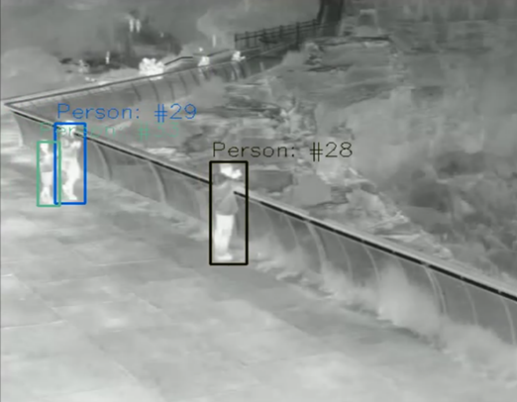
Pedestrians Tracking using Thermal Cameras. Individuals are assigned a unique ID which persists until they leave the camera view.

As with the RGB color camera recordings, following pedestrian detections, the DeepSORT [42] algorithm is applied to track multiple individuals in the thermal footage. An ID is assigned to each individual who is then tracked through subsequent video frames, as illustrated in Figure 11.

### Challenges and Future Directions

This above section described the computer vision pipeline which will be used to identify behaviors associated with crisis incidents. However, several challenges remain which require further consideration.

While previous studies have examined footage from metro settings, our study location is a very large outdoor space which experiences large variations in lighting and weather conditions. This can have a large impact on the performance of the detection algorithms, to the extent that RGB color cameras provide no footage for unlit areas at night. Furthermore, as a significant proportion of incidents occur after dark, the use of footage from thermal cameras is critical. However, thermal cameras provide less visual information that can be used in vision-based analysis. They also typically have a much lower resolution, resulting in additional challenges for pedestrian detection and tracking, and also suffer from blooming (blurred borders and outlines), and thus may not be suitable for pose estimation approaches that require identification of human limbs.

Therefore, a different strategy for identifying individual in crisis may need to be used using thermal cameras. For example, if the human coding study confirms that some individuals in crisis crouch down to leave possessions on the ground (a behavior which can be detected using pose estimation), or pace back and forth from the fence (a behavior that could be detected using trajectory analysis, and not requiring pose estimation), a thermal camera may only be able to detect the latter crisis behavior. Given that behaviors which rely on location—and not limb—analysis have been identified as key crisis behaviors in previous papers [8,9], this approach alone is likely to provide good accuracy in identifying individuals in crisis.

While thermal cameras provide less information for computer vision algorithms, thermal cameras of sufficient quality and minimal distance may still be able to identify behaviors through pose estimation. However, this requires experimental testing of the computer vision algorithm and possible postprocessing to improve the clarity of the image.

It is also important to consider the diversity of camera angles and distances to pedestrians within the scenes. These angles and distances are different for each camera at the site, and these may differ considerably from those which have been used to pretrain the selected algorithms, thus negatively affecting performance. Further annotations and fine-tuning may therefore be required for each scene included in the analysis.

Our current implementation uses a supervised learning approach to train models to recognize certain unit actions, such as walking, standing, waving, and taking a photo. However, a substantial human time effort is required to annotate these behaviors across video frames. It may be possible to incorporate unsupervised as well as active learning methods in conjunction with transfer learning to optimally leverage the existing annotated data without requiring additional manual annotations. To detect and differentiate between longer and more complex activities, higher-level reasoning may be required to aid the decision-making process. Not only will unit actions be detected, but also our system will learn movement patterns to allow the identification of individuals who appear to be in crisis. These results will be reported elsewhere upon the conclusion of data collection.

In addition to the technological aspects identified in this review, it is also important to assess the acceptability of this approach with decision makers, people with lived experience, and other stakeholders. For example, the Joint Commission in the United States released a statement prohibiting the use of video monitoring of patients with high suicide risk unless in-person monitoring is also present [47]. However, the statement notes that this decision is due to inability to immediately intervene with video monitoring alone. Therefore, future studies will also need to understand the concerns surrounding implementing such technologies, as well as how to address them.

### Conclusions

This paper has reviewed the existing literature pertaining to the use of CCTV and video data in understanding and preventing suicide. The narrative review found that studies have reported on 3 broad approaches: assessing depression risk by facial cues as a suicide risk factor; using CCTV and video to understand suicide after the fact through agonal sequences and determining suicidal intent; and finally using CCTV as a suicide prevention initiative. Studies have reported systems to generate alerts when a suicide attempt is detected, for example detecting hanging or jumping from a bridge; however these focus on the period after an attempt is made. Others have reported human coding studies to describe behaviors which can be observed in individuals during crisis prior to a suicide attempt. Building on these previously reported research themes, we reported progress on a current project to identify behaviors associated with crisis incidents at a specific site. The proposed methodology for a human annotation study is described, along with the development of a computer vision processing pipeline. We describe several challenges and considerations which are required to extend computer vision techniques to different settings. Ultimately, this research aims to automatically detect behaviors which may be indicative of a suicide attempt, allowing earlier intervention to save lives.

## Abbreviations

CCTV: closed-circuit television
LDC: linear discriminant classifier
PTZ: pan, tilt, and zoon
RGB-D: red, green, blue—depth
